# Famous Poisoners in Fiction

**Published:** 1894-04-14

**Authors:** 


### Famous Poisoners in Fiction.

### IX.—THE FAVOURITE POISONS AMONGST AUTHORS AND DRAMATISTS.

### Part I.

We must poison our heroine off, that is imperative, says the author to his "bosom" friend to whom he is confiding a sketch of his plot.

"By Jove, whatever poison will you use?" cries his listener.

"Oh, anything—arsenic most likely; it is the most popular one, and the best known," is the answer.

On the whole, the reply would be a right one, for in most of the famous trials for murder arsenic has been the weapon employed.

Madeline Smith, and nearer our own times, Mrs. Maybrick, were both accused of poisoning by arsenic, and this recalls to memory that by a strange coincidence, a well-known novel by Helen Mathers was appearing in serial parts at the very time that the trial of the latter was attracting intense attention, of which the complicated plot turned on arsenic poisoning. But with those novelists who have studied poison lore the famous Aqua Tofana is the favourite poison. This clear, cool water, with naught to show its deadly power, holds a position in romance which is more than its due, for since the day of its inventor it has been comparatively little used, or at least its use has been very seldom discovered, but then deaths from poisoning wrought by means of this subtle poison are very difficult of discovery. This famous Acquetta di Napoli, or Aqua Tofana as it is more usually called, was the favourite poison of Tophana, a woman who, in the seventeenth century, is suspected of having poisoned by its means over seven hundred people. This Aqua Tofana was a solution of arsenic, as was also the equally well-known Aqua de Perugia. The latter is said to have been prepared by killing a pig, disjointing it, and preparing it as for cooking, only using arsenic instead of salt. The juice or gravy thus prepared is far more poisonous than is arsenic in an ordinary form. A young disciple of Tophana, Hieronyna Spara by name, formed an association of young married women, one of the objects of which was "the assasination of their husbands." To quote the words of Delibard, the arch fiend hero of Lytton's "Lucretia," used in reference to this very association: "It was the Saturnalia of the weak—a burst of mocking licence against the strong; it was more, it was the innate force of the individual waging war against the many. In that age husbands were, indeed, lords of the household; they married mere children for their lands; they neglected and betrayed them; they were inexorable if the wife committed the faults set before her for example. Suddenly the wife found herself armed against her tyrant. His life was in her hands!"

Whilst on this subject, the fact should be noted that one of the theories given by a clever student of Indian history for the institution of Suttee in the East was that it was instituted to act as a check to an epidemic of wholesale husband poisoning which broke out once upon a time in Hindustan. I think men forget that they are entirely in their wives' hands when, for the sake of a pretty face, they carelessly place their very lives oftimes in the keeping of a perfect stranger, and, fascinated by her beauty, yield themselves captive to a nineteenth century Delilah.

Most novelists are rather vague with regard to the poisons with which they deal out death to the fictitious victims of a fictitious villain. They write up a fine scene in which the hero or heroine dies after making a magnificent speech, or whilst smiling benignly and forgivingly on the murderer. These idealists quite forget that in real life it would be far more probable that after the amount of poison taken by their heroes they would be rolling in agony on the ground with distorted face and clutched hands; but then the probable is never so far as from the realms of fiction. Dumas, however, is an exception to the rule, for he so minutely describes the working of brucine in his "Monte Cristo," that he teaches his readers much about a valuable medicine as well as poison; and in his "Diary of a late Physician," Warren also enters with minutiæ into the subject of poison lore.

> To merit heaven
> By making earth a hell.

has been the principle of others besides Torquemada and his followers.

Bartello, the old world Italian novelist who lived in the sixteenth century, introduced poison largely into some of his dramatic novels, and it naturally forms the chief subject, as the titles imply, in the famous French novels "L'empoisonneur," by L. Ninon, and "Les Empoisonneurs," by Abbé Guadet. Both these novels have a foundation of fact, being based on two famous trials for poisoning, one held at the beginning of the century, the other a little later, and both proving that the depravity of man is far beyond the imagination to conceive. This is also the case with Dumas' book, entitled "La Marquise de Brinvilliers," which is so true to the actual facts as to be unworthy the name of romance.

Alas! in describing beauty, nobility, self-sacrifice, and heroic deeds, the imagination soon outstrips the grandest earthly reality in an unattainable ideal, but when it comes to describing the acts of darkness and the perverseness of the human heart, the novelist finds himself culling from actual facts cruelties and infamy his imagination could not have invented or his mind stooped to believe.

But, partly because of fear of being caught tripping in this scientific age, and partly because the weapon of poison has a dastardliness English people at least abhor, and a meanness to which they will not lower themselves even in imagination, poison figures but little in the present day novel, and its position in the pages of romance is becoming less and less and beautifully less, except in those of the shilling shocker, and even then it is of the simplest description, laudanum or chloroform usually. Thus it comes to pass that poison lore need not be added to the novelists "chest of tools." The subject is, nevertheless, a keenly interesting one, and is also one greatly studied in the East, where, to quote the words of a new writer, "a *coup d'état* means an invitation to dinner with arsenic in the coffee served at dessert." In nearly all Eastern novels this great factor of Oriental life is introduced. Somehow, the subtilty of the working of poison, that dark, secret foe against which it is impossible to strike, seems in keeping with the serpentine characteristics of the mind of the Nabobs and sages of the East. How much it influenced the political and social European life in "ye olden time," men scarcely realise; but a historical novel not dealing with the use of poison would omit an important element of the life that was.

